# Automated vessel density detection in fluorescein angiography images correlates with vision in proliferative diabetic retinopathy

**DOI:** 10.1371/journal.pone.0238958

**Published:** 2020-09-11

**Authors:** Mohammad H. Bawany, Li Ding, Rajeev S. Ramchandran, Gaurav Sharma, Charles C. Wykoff, Ajay E. Kuriyan

**Affiliations:** 1 University of Rochester School of Medicine and Dentistry, Rochester, New York, United States of America; 2 Department of Electrical and Computer Engineering, University of Rochester, Rochester, New York, United States of America; 3 Department of Ophthalmology, University of Rochester Medical Center, Rochester, New York, United States of America; 4 Retina Consultants of Houston, Houston, Texas, United States of America; 5 Blanton Eye Institute, Houston Methodist Hospital & Weill Cornell Medical College, Houston, Texas, United States of America; 6 Retina Service, Wills Eye Hospital, Philadelphia, Pennsylvania, United States of America; 7 Center for Visual Science, University of Rochester, Rochester, New York, United States of America; University of Florida, UNITED STATES

## Abstract

**Purpose:**

To investigate the correlation between quantifiable vessel density, computed in an automated fashion, from ultra-widefield fluorescein angiography (UWFFA) images from patients with proliferative diabetic retinopathy (PDR) with visual acuity and macular thickness.

**Methods:**

We performed a secondary analysis of a prospective randomized controlled trial. We designed and trained an algorithm to automate retinal vessel detection from input UWFFA images. We then used our algorithm to study the correlation between baseline vessel density and best corrected visual acuity (BCVA) and CRT for patients in the RECOVERY study. Reliability of the algorithm was tested using the intraclass correlation (ICC). 42 patients from the Intravitreal Aflibercept for Retinal Non-Perfusion in Proliferative Diabetic Retinopathy (RECOVERY) trial who had both baseline UWFFA images and optical coherence tomography (OCT) data were included in our study. These patients had PDR without significant center-involving diabetic macular edema (central retinal thickness [CRT] ≤320μm).

**Results:**

Our algorithm analyzed UWFFA images with a reliability measure (ICC) of 0.98. A positive correlation (r = 0.4071, p = 0.0075) was found between vessel density and BCVA. No correlation was found between vessel density and CRT.

**Conclusions:**

Our algorithm is capable of reliably quantifying vessel density in an automated fashion from baseline UWFFA images. We found a positive correlation between computed vessel density and BCVA in PDR patients without center-involving macular edema, but not CRT.

**Translational relevance:**

Our work is the first to offer an algorithm capable of quantifying vessel density in an automated fashion from UWFFA images, allowing us to work toward studying the relationship between retinal vascular changes and important clinical endpoints, including visual acuity, in ischemic eye diseases.

## Introduction

Detection and monitoring of retinal ischemia have the potential to play an important role in the management and prognosis of patients with diabetic retinopathy and a number of other vision-threatening pathologies, including retinal vein occlusion and sickle cell retinopathy [1–4]. Prior to any visible vascular changes, diabetic retinopathy patients develop thinning of specific retinal layers as seen on spectral domain optical coherence tomography (OCT) images [5, 6]. Later, vascular changes are evident and histopathologic studies show microvascular occlusions in retinal capillaries [7, 8]. Further, irreversible damage and occlusion of macular capillaries has been shown to cause their dropout, leading to ischemia. Ischemia in the macula is especially concerning for vision loss. A cross-sectional study in diabetic patients found higher rates of diabetic macular ischemia (DMI) in more severe stages of diabetic retinopathy [3]. Reduced visual acuity has been seen in eyes with moderate to severe DMI [3].

Two imaging modalities are currently used to visualize blood flow in retinal vessels: fluorescein angiography (FA) and OCT angiography (OCT-A). Quantifying retinal vascular changes using these modalities is important to reproducibly compare longitudinal changes in the retinal vasculature of patients, to evaluate the impact of treatments on retinal vasculature, and to improve our understanding of the relationship between retinal vasculature and clinical outcomes, such as visual acuity and macular edema. Studies using quantitative vasculature data from OCT-A have found a positive correlation between macular vessel density and visual acuity in diabetic patients [9–11]. Studies are equivocal on the correlation between vessel density and macular thickness. A study of eyes with diabetic macular edema, found that lower macular vessel density correlated with greater macular thickness [12]. Another study found that in diabetic patients with no diabetic retinopathy (DR) or mild non-proliferative diabetic retinopathy (NPDR), the loss of vessel density was associated with macular ganglion cell layer thinning on OCT [13]. Similarly, another study in diabetic patients with and without retinopathy found a positive correlation between vessel density and central retinal thickness (CRT) [14].

In clinical applications, as compared to OCT images, ultra-widefield fluorescein angiography (UWFFA) images are currently assessed qualitatively. For research studies, the quantification of UWFFA has been focused on manually measuring the absence of retinal vessels or retinal non-perfusion and calculating an ischemic or nonperfusion index [15–18]. However, limitations of this labor-intensive approach include the time required for manual measurement and the impact of contrast on accurate vessel detection [19]. Despite these limitations, one study using this approach has found ischemic index in the mid-periphery was negatively associated with CRT and macular volume in patients with diabetic macular edema (DME) [20].

Currently, no widely accepted automated techniques exist to quantify retinal vascular changes in UWFFA similar to those employed in OCT-A. Such an automated technique for UWFFA would provide the ability to study longitudinal changes in vessels and relationships between vessel density and clinical endpoints, such as visual acuity and CRT. Our group has worked to address this unmet need by developing an automated algorithm that can detect retinal vasculature on UWFFA using a deep learning approach [21].

In this study, we utilized our automated algorithm to examine the relationship between vessel density, estimated via automated analysis, and best corrected visual acuity (BCVA) and CRT at the baseline visit of patients enrolled in the RECOVERY trial [22]. This trial is studying the effect of intravitreal aflibercept on retinal capillary non-perfusion in patients with proliferative diabetic retinopathy without macular edema. Our overall goal is to investigate how quantifiable vascular changes from UWFFA images can be used to predict clinically meaningful endpoints. For this specific study, we focused on evaluating only the macular vessel density to determine if we can reproduce relationships found in OCT-A studies between macular vessel density and BCVA and CRT, using our automated tools for UWFFA.

## Methods

### Study subjects and data

Patients with proliferative diabetic retinopathy without center-involving macular edema (defined as central subfield thickness >320 μm) who were enrolled in the RECOVERY trial were included in our study [22]. The RECOVERY trial assessed the safety and efficacy of intravitreal aflibercept injections given either every 4 weeks or every 12 weeks for the treatment of retinal capillary non-perfusion. Primary objectives of the RECOVERY study were to measure treatment-related adverse events and change in non-perfusion. Secondary outcomes included assessments of changes in visual acuity, changes in area of macular capillary non-perfusion as seen on UWFFA images, and changes in area of retinal capillary non-perfusion outside of the macula. For the current study, patients with complete baseline UWFFA images and visual acuities were included (n = 42).

Sterling Institutional Review Board/Ethics Committee approval was obtained for the enrollment, study, and treatment of patients in this Health Insurance Portability and Accountability Act (HIPAA)-compliant trial adhering to the tenets of the Declaration of Helsinki. Data were collected at Retina Consultants of Houston (Houston, Katy, and Woodlands, Texas). De-identified images and corresponding clinical data were transmitted to the University of Rochester for the analysis described in the current study. The University of Rochester Research Subject Review Board approved the current study and determined that the secondary analysis of de-identified images conducted is not research involving human subjects as defined by Department of Health and Human Services and Federal Drug Administration regulations.

UWFFA images for each patient were acquired using the Optos California and 200Tx cameras (Optos, Marlborough, MA, USA) [23]. Using software available from the manufacturer, UWFFA images were then transformed into stereographic projection images to create standard montage images for our analysis. Visual acuity was tested using Early Treatment of Diabetic Retinopathy Study (ETDRS) letters. The Heidelberg Spectralis OCT (Heidelberg Engineering, Inc., Heidelberg, Germany) was used to determine the CRT.

### Vessel detection and quantification

**Fig 1:**
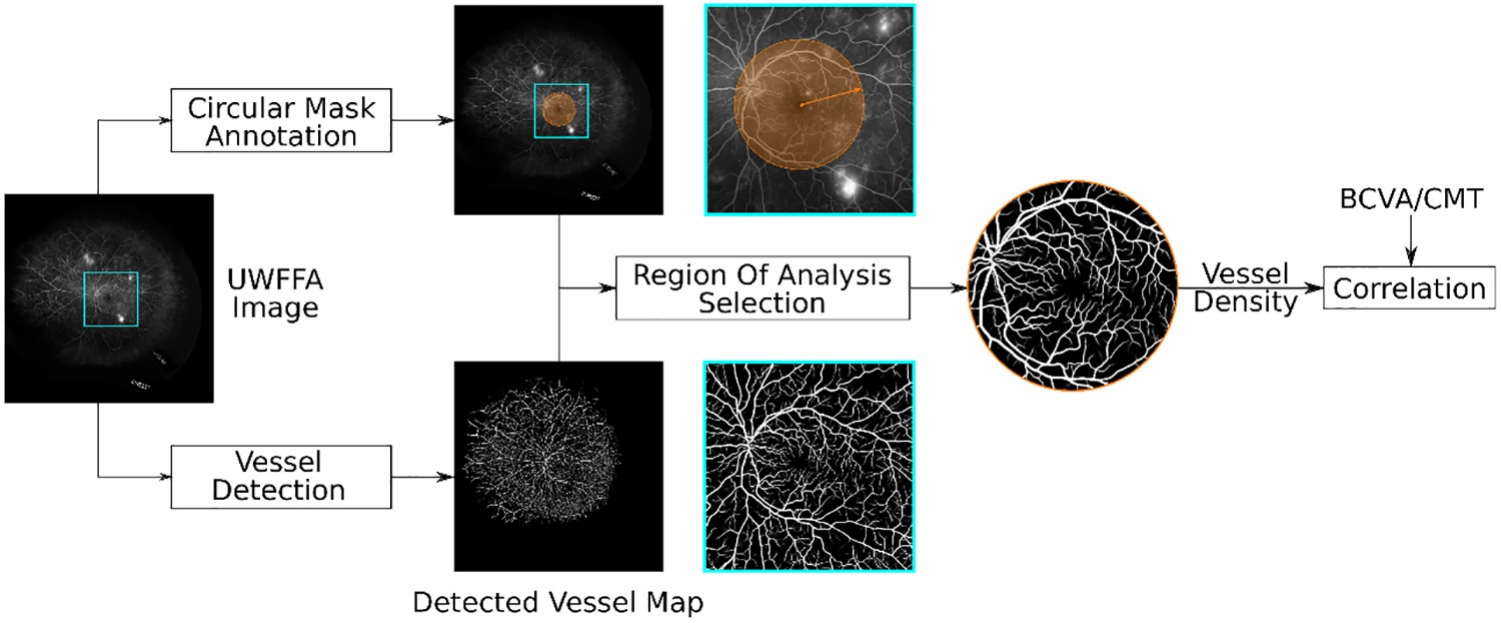
Vessel density computation from UWFFA images using our novel fully-automated algorithm. A deep neural network is trained to generate a vessel map by segmenting retinal vessels from input ultra-widefield fluorescein angiography (UWFFA) images. From the vessel map, vessel density is automatically estimated within a circular region around the fovea. The output vessel density can then be correlated to best corrected visual acuity (BCVA) and central retinal thickness (CRT).

The proposed framework for correlating vessel density with BCVA and CRT is depicted in Fig 1. As shown in the lower branch in Fig 1, retinal blood vessels are detected using a deep neural network organized in the U-Net architecture [24] that has been extensively utilized for medical image segmentation. The network was trained on a dataset consisting of 8 UWFFA images and the corresponding binary ground truth vessel maps using a novel human-in-the-loop procedure that reduces the burden of annotation [25]. The trained network achieved the area under the Precision-Recall curve of 0.930. Additional, technical details can be found in an independent publication [21], parts of which have been reported in preliminary form [19, 26, 27]. We apply the trained deep neural network to the (baseline) UWFFA images used in this study. The output from the deep neural network is a vessel map where pixel intensity, ranging from 0 to 1, indicates the probability of a corresponding pixel being a vessel. The probabilistic vessel map is then converted into the binary representation by setting a threshold of 0.5, which is shown to be close to the optimal threshold [21].

The top branch in Fig 1 shows the selection region over which the analysis is performed. For each UWFFA image, we manually annotated the center of the fovea. We chose the center of the fovea because it provides a consistent landmark that allows us to select the same region of interest for analysis across different participants. Circular regions centered at the fovea are created for computing the vessel density, which is estimated as the ratio of the number of vessel pixels to the total number of pixels within the circular area. The vessel density was estimated for circular regions centered on the fovea. pixels. The step size is 1 pixel, which translates to 540 circular regions with radii ranging from 10 to 550 pixels. The physical dimensions corresponding to the pixel measurements were determined by using the OptosAdvance software [28].

To measure the reliability of the automated vessel detection technique, we chose, for each patient, two consecutive UWFFA images that were captured after the vessels were fully perfused. Vessel density within a common circular region containing the macula was estimated for each pair of UWFFA images using the automated technique. For the reliability test, we chose the circular region around the fovea with the radius of 550 pixels, which is the largest region of analysis for the study thus far. The reliability test is currently limited to this circular region and future studies will aim to analyze the peripheral regions of the UWFFA. Note that the reliability analysis requires more than one FA image for each patient, which were selected from the original FA frames without the stereographic projection into the standard montage.

### Statistical analysis

For each radius, the Spearman rank correlation coefficient [29] was computed between the estimated vessel density and the BCVA reported as per the ETDRS letters. The correlation coefficient takes values from -1 to +1. Values of +1 and -1 indicate perfect positive and perfect negative monotonic correlations between the ranks, respectively, and a value of 0 represents no correlation. A statistical significance test was performed; the null hypothesis is that no correlation exists between the vessel density and BCVA. A p-value less than 0.05 is considered statistically significant. The radius at which the computed correlation coefficients showed minimum variability (assessed by repeatedly computing correlation coefficients leaving out data for one patient and computing the standard deviation of the resulting 42 values) was used for further analysis. Correlation between the estimated vessel density and the CRT was similarly computed and analyzed for statistical significance. The reliability of estimating vessel density was assessed by visualizing the inter-sample agreement using Bland-Altman plots and quantified by computing the intraclass correlation coefficient (ICC) [30].

## Results

**Fig 2:**
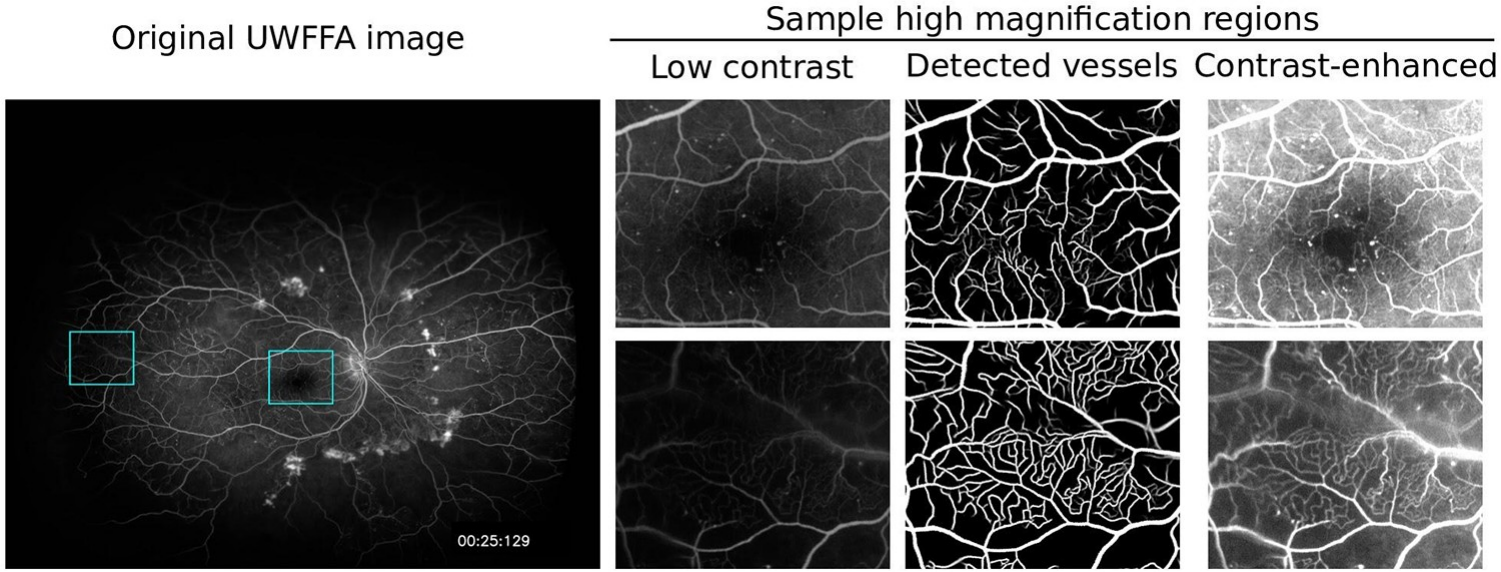
Sample results of vessel detection in UWFFA images using our automated algorithm show that the algorithm is able to detect vessels that are difficult to identify manually in low-contrast images. From left to right: original ultra-widefield fluorescein angiography (UWFFA) image, enlarged views of rectangular regions that have low contrast, and the corresponding automatically detected vessel maps, and contrast-enhanced views. Our automated detection algorithm detects fine vessels that are difficult to manually identify in low contrast views and are verified as actual vessels by the contrast enhanced views.

Forty-two patients from the RECOVERY trial were included in our study. Statistics of the baseline demographics and clinical data of these patients are reported in Table 1. Examples of vessel maps detected from UWFFA images using the automated technique are shown in Fig 2. These examples highlight the challenge of contrast variations in FA images and the advantage that automated detection provides. The highlighted rectangular regions in the leftmost image in Fig 2 contain many fine vessels that are difficult to identify in the original UWFFA images. Our automated detection algorithm, however, is not affected by changes in image contrast and is able to detect these vessels [21]. Careful contrast enhancement of these regions validates that the fine vessels are actually present. The automated vessel detection can be performed quickly to allow it to be integrated into the clinical workflow; mean time for automated vessel detection from a UWFFA image is 22.05 seconds (standard deviation 0.21 seconds).

**Table 1:** Baseline demographics and clinical data of patients from the RECOVERY trial included in the current study.

	Study Patients (n = 42)
Mean age, years (median, IQR)	47.8 (47, 42–56)
Female, n (%)	20 (48)
OD Study Eye, n (%)	21 (50)
Mean HbA1c, % (median, IQR)	9.48 (8.9, 7.5–10.5)
BCVA (ETDRS letters), mean (SD)	79 (7.6)
Macula thickness, mean μm (SD)	278 (31)

BCVA = best corrected visual acuity, ETDRS = Early Treatment Diabetic Retinopathy Study, HbA1c = hemoglobin A1c, IQR = inter-quartile range, OD = right eye, SD = standard deviation.

**Fig 3:**
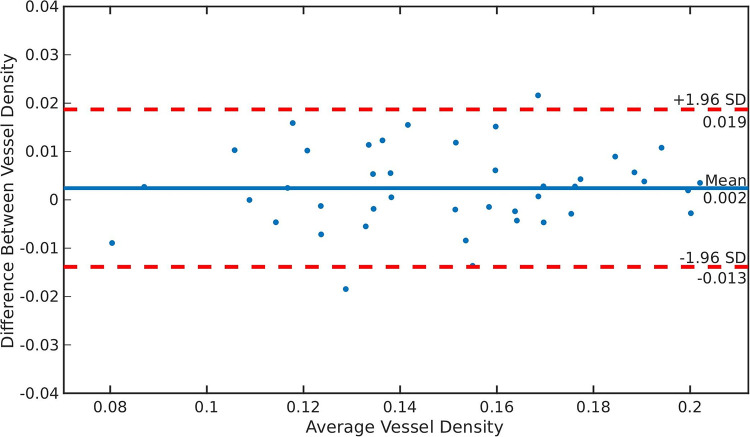
Bland-Altman plot indicating the reliability of using our algorithm for vessel density estimation from UWFFA images. The Bland-Altman plot illustrates the agreement between vessel density estimates from two consecutively acquired ultra-widefield fluorescein angiography (UWFFA), with a mean difference between vessel density of only 0.002 (standard deviation 0.008).

Fig 3 shows the Bland-Altman plot that illustrates the agreement between vessel density estimates from the two different captures. The mean difference between vessel density was 0.002 (standard deviation 0.008). The ICC is computed by a 2-way mixed-effects model, average measures, and absolute agreement [30]. A high ICC of 0.980 (95% confidence interval: 0.963 to 0.990) was obtained that indicates excellent reliability.

**Fig 4:**
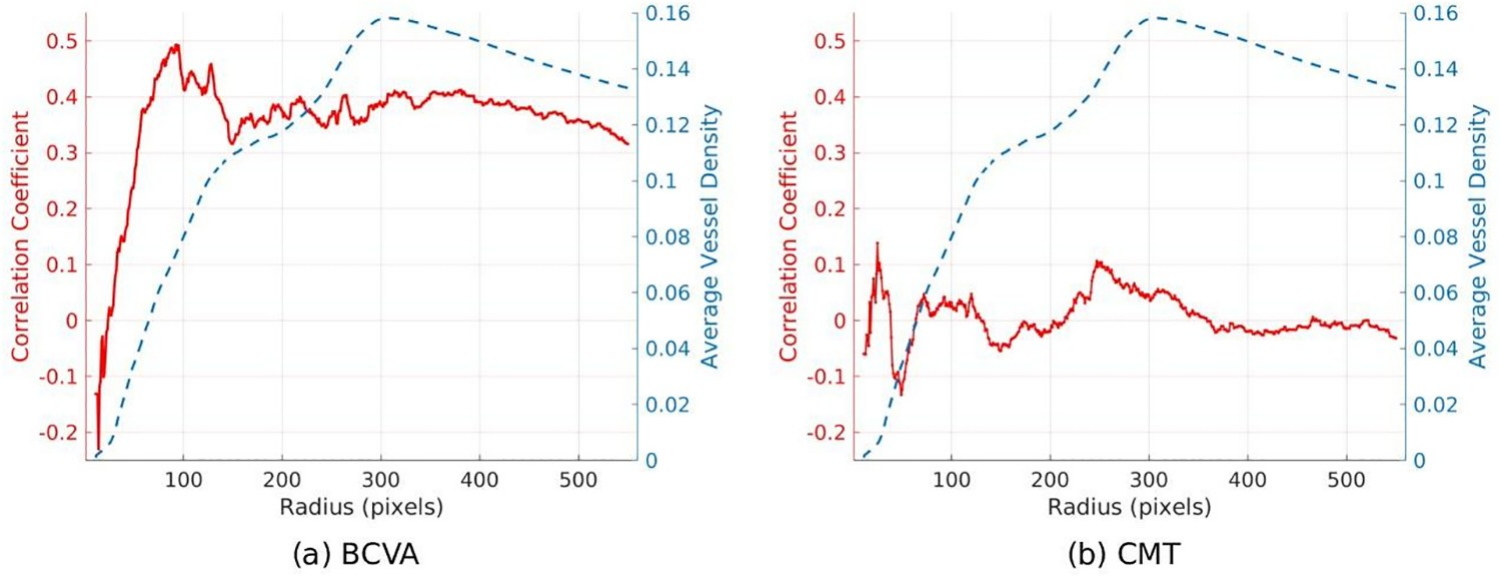
Average vessel density and correlation with best corrected visual acuity (BCVA) and central retinal thickness (CRT) for circular regions with varying radii. Vessel density shows correlation with BCVA but not with CRT. A. Plot of correlation coefficients of BCVA (solid red line) with vessel density (dashed blue line) computed from circular pixel regions with radii ranging from 10 to 550 pixels centered on the fovea. There were significant positive correlations between vessel density and BCVA for radii larger than 53 pixels (p<0.05). B. Plot of correlation coefficients of CRT (solid red line) with vessel density (dashed blue line) computed from circular pixel regions with radii ranging from 10 to 550 pixels centered on the fovea. No significant correlation was found between vessel density and CRT for any radii.

Fig 4 shows the correlation coefficients and mean vessel density as a function of the circular radius, in pixels, used for the analysis. We found significant positive correlations between vessel density and BCVA for radii larger than 53 pixels (p<0.05), indicating that higher vessel density is associated with better vision. No significant correlation was found between vessel density and CRT.

**Fig 5:**
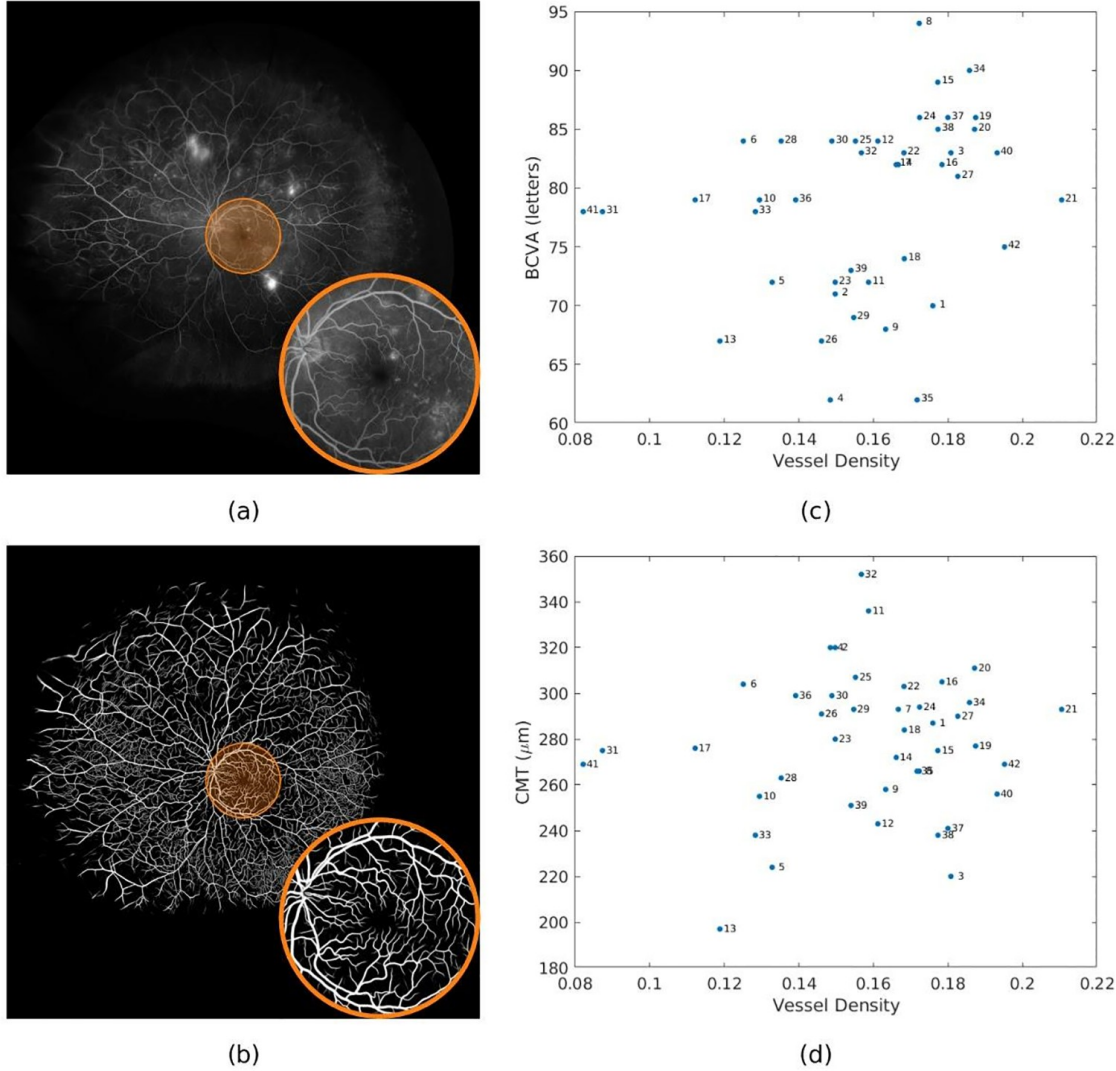
Vessel density within circular posterior pole region of radius 315 pixels (approximate diameter of 11.05 mm) correlates with best corrected visual acuity (BCVA), but not central retinal thickness (CRT). A. Example of ultra-widefield fluorescein angiography (UWFFA) image with the circular posterior pole region of radius 315 pixels (approximate diameter 11.05 mm) highlighted as an orange circle. B. Example of detected vessel map for the circular region. C. Scatter plots of vessel density vs. BCVA for the 42 patients in the study demonstrating a significant positive correlation between vessel density and BCVA (r = 0.4071, p = 0.0075). D. Scatter plots of vessel density vs. CRT for the 42 patients in the study, demonstrating absence of significant correlation between vessel density and CRT (r = 0.0533, p = 0.7376).

**Fig 6:**
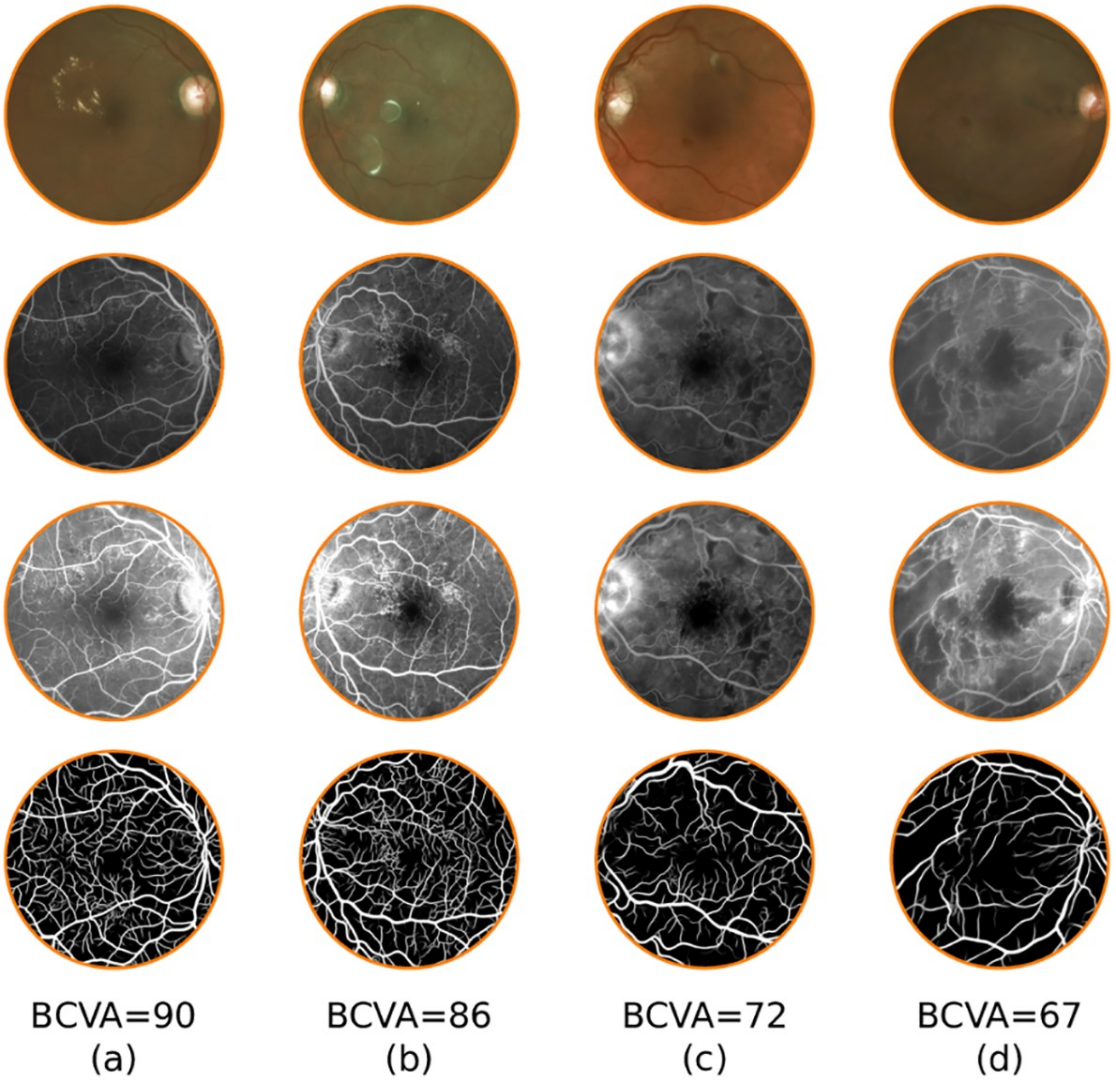
Example images across the range of best corrected visual acuity (BCVA) seen in the study demonstrating differences in vessel density. From top to bottom: fundus image, original UWFFA, contrast-enhanced UWFFA views, and detected vessel maps for four patients over circular regions centered at the fovea. Example images in columns (a) and (b) have better BCVA and higher vessel densities, while those in columns (c) and (d) have worse BCVA and lower vessel densities.

The correlation coefficients between the vessel density and BCVA showed minimum variability for radius of 315 pixels, which was chosen for presentation of further results and analysis. Fig 5 shows the circular mask with a radius of 315 pixels overlaid on a sample UWFFA image (Fig 5a) and the corresponding detected vessel map (Fig 5c). Scatter plots between vessel density and BCVA, and between vessel density and CRT, are shown in Fig 5b and 5d, respectively. For this mask, the correlation coefficients of BCVA and CRT are 0.4071 (p = 0.0075) and 0.0533 (p = 0.7376), respectively. Using the Optos *Advance* software [28] we found that masks with a radius of 315 pixels corresponded to a diameter of approximately 11.05 mm (standard deviation 0.12 mm). Detected vessel maps from UWFFA regions of two patients who have high BCVA and two patients who have low BCVA are shown in Fig 6. Patients with worse BCVA exhibited lower vessel densities.

## Discussion

Current literature on OCT-A image analysis has shown positive correlations between macular vessel density and visual acuity. The objective of our study was to demonstrate that quantitative estimates of vessel density obtained from automated vessel extraction in UWFFA, a modality that has only been studied qualitatively in the past, similarly exhibits a positive correlation with visual acuity and is consistent with the OCT-A based finding. Replication of the finding based on automated UWFFA image analysis potentially opens the path for greater use of quantitative analysis based on UWFFA images. UWFFA is the current method of choice for assessing far peripheral retina diabetic ischemia, and, to our knowledge, we are the first group to offer an algorithm capable of quantifying vessel density in an automated fashion from UWFFA images, allowing us to work toward our goal of automating peripheral retinal vessel evaluation.

Additional strengths of our algorithm include its high reliability, and its ability to automatically detect vessels in image regions in the presence of significant variations in the contrast between the background and the vasculature, variations which are ubiquitous in UWFFA imagery. While the detected vessels can be seen manually via a process of repeatedly adjusting contrast and viewing different regions, the tedium and time requirement for doing this are prohibitive in clinical practice.

As a way to limit the amount of manual adjustments, a number of prior studies have focused on only the foveal avascular zone (FAZ) as a potential metric to screen for and track ischemic eye diseases. Studies using both qualitative and manual quantification methods have shown that the FAZ can enlarge and become irregular as ischemic diseases, such as DR, advance [31–35]. One study examining FA images for a link between FAZ and visual function found no relationship between FAZ area and visual acuity in mild grades of ischemia, but found worse vision in patients with moderate to severe ischemia who had larger FAZ areas [3]. A few studies, however, indicate that FAZ interpretation has diagnostic limitations—the FAZ can be highly variable within control groups and among healthy subjects [10]. It is also suggested that parafoveal retinal tissue is able to function normally without direct retinal blood supply [36].

Vessel density in the entire macula is being explored as a promising new metric to measure the extent of disease in eyes affected by DR and other conditions that can alter retinal vasculature. Current commercially available OCT-A machines are generally used to image the macula. Studies have shown, using OCT-A, that patients with eye diseases, such as DR, have lower macular vessel densities than healthy controls [37–40]. Further, macular vessel density has been correlated with important clinical outcomes, such as visual acuity and macular thickness [9, 12, 34, 41].

Though OCT-A has been the imaging modality of choice in these studies due to its non-invasive nature, methods gauging vessel density using published sets of OCT-A images have differed significantly in their results. The algorithms used to calculate vessel density in OCT-A images vary; they may accompany instruments as proprietary software, or they may be made by research groups or companies to allow for post-image processing of exported images. As a result, the ensuing vessel density measurement of the same image sets or patients differ among the various devices, algorithms, scan locations, and scanned area sizes [42–45]. Moreover, studies comparing a variety of OCT-A-based algorithms to detect vessel density have found no single algorithm that outperforms others in all retinal plexuses examined [37]. Significant motion and segmentation artifacts have been noted when using the manufacturer’s OCT-A algorithms [46]. Overall, it has been found that methods that incorporate manual annotation of OCT-A images offer the best discrimination of retinal vessels across plexuses [37]. Manual annotation, however, is highly subjective, highly time-consuming, and further, requires trained graders.

However, macular vessel density has not been studied using FA due to the limited ability to quantify macular vessels using FA prior to this work. Using the approach described in this paper, we successfully demonstrated a relationship between visual acuity and macular vessel density, similar to that found in prior OCT-A studies. While OCT-A is becoming more widely adopted, FA, especially UWFFA, continues to play an important role in the assessment and management of diabetic retinopathy. Significant peripheral retinal pathology can be seen in diabetic patients, outside of the area examined by the ETDRS 7 standard fields [47, 48]. With advances in retinal imaging technology, UWF images can capture more than 80% of the retina for examination, leading to identification of additional diabetic lesions in the retina periphery [47, 49]. UWF has advantages over the ETDRS 7 fields assessment, in that it increases the frequency of identifying diabetic retinopathy, and decreases image acquisition and evaluation time [50, 51].

A computerized approach to detecting retinal vasculature, like ours applied to UWFFA images, provides the ability to quantify retinal vasculature more peripherally than swept-source OCT-A devices [52]. Importantly, our algorithm is not affected by changes in image contrast, which enables it to detect vessels that are not able to be visualized by manual vessel detection without extensive attention to adjusting the contrast in order to detect all the vessels present as highlighted in Fig 2 [21]. Future applications of the algorithm can potentially provide a quantitative assessment of retinal vasculature density in both the posterior pole and far periphery without montaging, providing additional data compared to current OCT-A machines. Furthermore, the algorithm can be applied to study longitudinal vascular density changes in vascular diseases, and to study how local and systemic treatments impact retinal vascular disease progression. Quantification of retinal vasculature can facilitate studying the relationship between vascular changes and important clinical endpoints, including visual acuity, in ischemic disease such as diabetic retinopathy, as shown in this study.

We have highlighted the clinical significance of our work by establishing a correlation between vessel density and visual acuity in PDR patients without significant center-involving macular edema (CRT >320 μm). Our finding of a correlation between vessel density and visual acuity is in agreement with that noted in a number of OCT-A studies, mentioned previously [9, 12, 34, 41]. Although previous studies have found that decreased vessel density correlates to increased macular thickness in patients with diabetic macular edema as opposed to healthy controls [12]. Vessel density does not correlate with CRT in our study population, likely because all of the patients in our study did not have significant center-involving macular edema due to the exclusion criteria for the RECOVERY study (CRT >320 μm). One limitation of our study is that we have used a small sample size to train our algorithm and to report our outcomes. Despite this limitation, we have developed a highly reproducible algorithm for automated vessel detection. Although we developed an algorithm that was capable of detecting far peripheral vessels, we focused on the posterior pole in this study to ensure that the algorithm produced findings consistent with previously published reports using OCT-A data.

Our future directions include studying the relationship with visual acuity and vessel density beyond the posterior pole at baseline as well as longitudinally during the course of the study. This will also enable us to assess the impact of intravitreal aflibercept on peripheral non-perfusion in a quantitative fashion. As a number of prior studies examining retinal vessel density in various disease states have concluded, though vessel density may offer a future surrogate endpoint for clinical trials, research is still needed to uncover its full efficacy in predicting clinical outcomes before it can be routinely incorporated into clinical practice [53].

## Supporting information

S1 Table Individual values of best corrected visual acuity (BCVA), central retinal thickness (CRT), and vessel density for circular regions with different radii used for the correlation analysis presented in Figs 4 and 5. The excel table provides the data used for computing the correlation coefficients between estimated vessel density and BCVA/CRT. Specifically, the file includes header and individual rows for each patient that provide the following columns of information: ID Number: participant serial number; BCVA: baseline BCVA value (ETDRS letters); CRT: baseline CRT value in micrometers; VD@R = Value: vessel density estimated from the circular region with radius R pixels, i.e. from pixels (x,y) such that (x-X0)^2^+(y-Y0)^2^ ≤ R. This table provides the baseline best-corrected visual acuity, central retinal thickness, and vessel density values at all the different diameter circles centered on the fovea that were measured for each patient.
(XLSX)

## Abbreviations

BCVA: best corrected visual acuity
CRT: central retinal thickness
DME: diabetic macular edema
DMI: diabetic macular ischemia
DR: diabetic retinopathy
ETDRS: Early Treatment of Diabetic Retinopathy Study
FA: fluorescein angiography
FAZ: foveal avascular zone
ICC: intraclass correlation
NPDR: non-proliferative diabetic retinopathy
OCT: optical coherence tomography
OCT-A: optical coherence tomography angiography
PDR: proliferative diabetic retinopathy
UWFFA: ultra-widefield fluorescein angiography

## References

[1] Lee CM, Charles HC, Smith RT, Peachey NS, Cunha-Vaz JG, Goldberg MF. Quantification of macular ischaemia in sickle cell retinopathy. *Br J Ophthalmol*. 1987;71(7):540–545. 3651368 10.1136/bjo.71.7.540 PMC1041222

[2] Khayat M, Williams M, Lois N. Ischemic retinal vein occlusion: characterizing the more severe spectrum of retinal vein occlusion. *Surv Ophthalmol*. 2018;63(6):816–850. 29705175 10.1016/j.survophthal.2018.04.005

[3] Sim DA, Keane PA, Zarranz-Ventura J, et al The Effects of Macular Ischemia on Visual Acuity in Diabetic Retinopathy. *Investig Opthalmology Vis Sci*. 2013;54(3):2353–2360. 10.1167/iovs.12-11103 23449720

[4] Hayreh SS, Klugman MR, Beri M, Kimura AE, Podhajsky P. Differentiation of ischemic from non-ischemic central retinal vein occlusion during the early acute phase. *Graefes Arch Clin Exp Ophthalmol Albrecht Von Graefes Arch Klin Exp Ophthalmol*. 1990;228(3):201–217. 10.1007/BF00920022 2361592

[5] Park HY-L, Kim IT, Park CK. Early diabetic changes in the nerve fibre layer at the macula detected by spectral domain optical coherence tomography. *Br J Ophthalmol*. 2011;95(9):1223–1228. 21216799 10.1136/bjo.2010.191841

[6] Verma A, Rani PK, Raman R, et al Is neuronal dysfunction an early sign of diabetic retinopathy? Microperimetry and Spectral Domain Optical Coherence Tomography (SD-OCT) Study in individuals with diabetes, but no diabetic retinopathy. *Eye*. 2009;23(9):1824–1830. 19648899 10.1038/eye.2009.184

[7] Ashton N. Vascular basement membrane changes in diabetic retinopathy. Montgomery lecture, 1973. *Br J Ophthalmol*. 1974;58(4):344–366. 4138036 10.1136/bjo.58.4.344 PMC1214780

[8] Schröder S, Palinski W, Schmid-Schönbein GW. Activated monocytes and granulocytes, capillary nonperfusion, and neovascularization in diabetic retinopathy. *Am J Pathol*. 1991;139(1):81–100. 1713023 PMC1886150

[9] Dupas B, Minvielle W, Bonnin S, et al Association Between Vessel Density and Visual Acuity in Patients With Diabetic Retinopathy and Poorly Controlled Type 1 Diabetes. *JAMA Ophthalmol*. 2018;136(7):721–728. 29800967 10.1001/jamaophthalmol.2018.1319 PMC6136049

[10] Arend O. The Relationship of Macular Microcirculation to Visual Acuity in Diabetic Patients. *Arch Ophthalmol*. 1995;113(5):610–614. 7748131 10.1001/archopht.1995.01100050078034

[11] Hwang TS, Gao SS, Liu L, et al Automated Quantification of Capillary Nonperfusion Using Optical Coherence Tomography Angiography in Diabetic Retinopathy. *JAMA Ophthalmol*. 2016;134(4):367–373. 26795548 10.1001/jamaophthalmol.2015.5658 PMC4978127

[12] Toto L, D’Aloisio R, Di Nicola M, et al Qualitative and Quantitative Assessment of Vascular Changes in Diabetic Macular Edema after Dexamethasone Implant Using Optical Coherence Tomography Angiography. *Int J Mol Sci*. 2017;18(6):1181. 10.3390/ijms18061181 PMC5486004 28574436

[13] Tang FY, Ng DS, Lam A, et al Determinants of Quantitative Optical Coherence Tomography Angiography Metrics in Patients with Diabetes. *Sci Rep*. 2017;7(1):2575. 28566760 10.1038/s41598-017-02767-0 PMC5451475

[14] Dodo Y, Suzuma K, Ishihara K, et al Clinical relevance of reduced decorrelation signals in the diabetic inner choroid on optical coherence tomography angiography. *Sci Rep*. 2017;7(1):5227. 28701715 10.1038/s41598-017-05663-9 PMC5507874

[15] Tan CS, Chew MC, van Hemert J, Singer MA, Bell D, Sadda SR. Measuring the precise area of peripheral retinal non-perfusion using ultra-widefield imaging and its correlation with the ischaemic index. *Br J Ophthalmol*. 2016;100(2):235–239. 26135013 10.1136/bjophthalmol-2015-306652

[16] Silva PS, Dela Cruz AJ, Ledesma MG, et al Diabetic Retinopathy Severity and Peripheral Lesions Are Associated with Nonperfusion on Ultrawide Field Angiography. *Ophthalmology*. 2015;122(12):2465–2472. 26350546 10.1016/j.ophtha.2015.07.034

[17] Wang K, Ghasemi Falavarjani K, Nittala MG, et al Ultra-Wide-Field Fluorescein Angiography–Guided Normalization of Ischemic Index Calculation in Eyes With Retinal Vein OcclusionUWFFA Guided Normalization of Ischemic Index in RVO. *Invest Ophthalmol Vis Sci*. 2018;59(8):3278–3285. 29971447 10.1167/iovs.18-23796

[18] Yu G, Aaberg MT, Patel TP, et al Quantification of Retinal Nonperfusion and Neovascularization With Ultrawidefield Fluorescein Angiography in Patients With Diabetes and Associated Characteristics of Advanced Disease. *JAMA Ophthalmol*. 2020;138(6):680–688. 10.1001/jamaophthalmol.2020.1257 32352506 PMC7193527

[19] Ding L, Kuriyan A, Ramchandran R, Sharma G. Multi-scale morphological analysis for retinal vessel detection in wide-field fluorescein angiography. In: 2017 IEEE Western New York Image and Signal Processing Workshop (WNYISPW). IEEE; 2017:1–5.

[20] Fan W, Wang K, Ghasemi Falavarjani K, et al Distribution of Nonperfusion Area on Ultra-widefield Fluorescein Angiography in Eyes With Diabetic Macular Edema: DAVE Study. *Am J Ophthalmol*. 2017;180:110–116. 28579062 10.1016/j.ajo.2017.05.024

[21] Ding L, Bawany MH, Kuriyan AE, Ramchandran RS, Wykoff CC, Sharma G. A Novel Deep Learning Pipeline for Retinal Vessel Detection in Fluorescein Angiography. *IEEE Trans Image Process*. 2020;29(1):6561–6573. 10.1109/TIP.2020.2991530 PMC7648732 32396087

[22] Wykoff CC, et al Intravitreal Aflibercept for Retinal Non-Perfusion in Proliferative Diabetic Retinopathy: Outcomes from the RECOVERY Randomized Trial. *Ophthalmol Retina*. 2019;3(12):1076–1086. 31542339 10.1016/j.oret.2019.07.011

[23] Optos California Tech Sheet. Published online 2015. https://www.optos.com/globalassets/www.optos.com/products/california/california-brochure.pdf

[24] Ronneberger O, Fischer P, Brox T. U-Net: Convolutional networks for biomedical image segmentation. In: International Conference on Medical Image Computing and Computer-Assisted Intervention. 2015:234–241.

[25] Ding L, Bawany MH, Kuriyan AE, Ramchandran RS, Wykoff CC, Sharma G. RECOVERY-FA19 dataset. Published online 2019. 10.21227/m9yw-xs04

[26] Ding L, Kuriyan A, Ramchandran R, Sharma G. Quantification of Longitudinal Changes in Retinal Vasculature from Wide-Field Fluorescein Angiography via a Novel Registration and Change Detection Approach. In: 2018 IEEE International Conference on Acoustics, Speech and Signal Processing (ICASSP). IEEE; 2018:1070–1074.

[27] Ding L, Kuriyan A, Ramchandran R, Sharma G. Retinal Vessel Detection in Wide-Field Fluorescein Angiography with Deep Neural Networks: A Novel Training Data Generation Approach. In: 2018 25th IEEE International Conference on Image Processing (ICIP). IEEE; 2018:356–360.

[28] Optos. Optos Software Products. Published 2019. Accessed July 28, 2019. https://www.optos.com/en/products/our-software-products/

[29] Gibbons JD, Chakraborti S. *Nonparametric Statistical Inference*. 4th ed Marcel Dekker; 2003.

[30] Koo TK, Li MY. A Guideline of Selecting and Reporting Intraclass Correlation Coefficients for Reliability Research. *J Chiropr Med*. 2016;15(2):155–163. 27330520 10.1016/j.jcm.2016.02.012 PMC4913118

[31] Garcia JMB de B, Lima TT, Louzada RN, Rassi AT, Isaac DLC, Avila M. Diabetic Macular Ischemia Diagnosis: Comparison between Optical Coherence Tomography Angiography and Fluorescein Angiography. *J Ophthalmol*. Published online 2016:1–6. 10.1155/2016/3989310 PMC5116522 27891250

[32] Early photocoagulation for diabetic retinopathy. ETDRS report number 9. Early Treatment Diabetic Retinopathy Study Research Group. *Ophthalmology*. 1991;98(5 Suppl):766–785. 2062512

[33] Casselholmde Salles M, Kvanta A, Amrén U, Epstein D. Optical Coherence Tomography Angiography in Central Retinal Vein Occlusion: Correlation Between the Foveal Avascular Zone and Visual Acuity. *Investig Opthalmology Vis Sci*. 2016;57(9):OCT242. 10.1167/iovs.15-18819 27409478

[34] Samara WA, Shahlaee A, Adam MK, et al Quantification of Diabetic Macular Ischemia Using Optical Coherence Tomography Angiography and Its Relationship with Visual Acuity. *Ophthalmology*. 2017;124(2):235–244. 27887743 10.1016/j.ophtha.2016.10.008

[35] Balaratnasingam C, Inoue M, Ahn S, et al Visual Acuity Is Correlated with the Area of the Foveal Avascular Zone in Diabetic Retinopathy and Retinal Vein Occlusion. *Ophthalmology*. 2016;123(11):2352–2367. 27523615 10.1016/j.ophtha.2016.07.008

[36] Bresnick GH, Condit R, Syrjala S, Palta M, Groo A, Korth K. Abnormalities of the foveal avascular zone in diabetic retinopathy. *Arch Ophthalmol Chic Ill 1960*. 1984;102(9):1286–1293. 10.1001/archopht.1984.01040031036019 6477244

[37] Rabiolo A, Gelormini F, Sacconi R, et al Comparison of methods to quantify macular and peripapillary vessel density in optical coherence tomography angiography. Cheung G, ed. PLOS ONE. 2018;13(10):e0205773. 30335815 10.1371/journal.pone.0205773 PMC6193681

[38] Kim AY, Chu Z, Shahidzadeh A, Wang RK, Puliafito CA, Kashani AH. Quantifying Microvascular Density and Morphology in Diabetic Retinopathy Using Spectral-Domain Optical Coherence Tomography Angiography. *Investig Opthalmology Vis Sci*. 2016;57(9):OCT362. 10.1167/iovs.15-18904 PMC4968771 27409494

[39] Zahid S, Dolz-Marco R, Freund KB, et al Fractal Dimensional Analysis of Optical Coherence Tomography Angiography in Eyes With Diabetic Retinopathy. *Investig Opthalmology Vis Sci*. 2016;57(11):4940–4947. 10.1167/iovs.16-19656 27654421

[40] Nesper PL, Roberts PK, Onishi AC, et al Quantifying Microvascular Abnormalities With Increasing Severity of Diabetic Retinopathy Using Optical Coherence Tomography Angiography. *Investig Opthalmology Vis Sci*. 2017;58(6):BIO307. 10.1167/iovs.17-21787 PMC5693005 29059262

[41] Yarmohammadi A, Zangwill LM, Diniz-Filho A, et al Relationship between Optical Coherence Tomography Angiography Vessel Density and Severity of Visual Field Loss in Glaucoma. *Ophthalmology*. 2016;123(12):2498–2508. 27726964 10.1016/j.ophtha.2016.08.041 PMC5362128

[42] Lei J, Durbin MK, Shi Y, et al Repeatability and Reproducibility of Superficial Macular Retinal Vessel Density Measurements Using Optical Coherence Tomography Angiography En Face Images. *JAMA Ophthalmol*. 2017;135(10):1092–1098. 28910435 10.1001/jamaophthalmol.2017.3431 PMC5710485

[43] Corvi F, Pellegrini M, Erba S, Cozzi M, Staurenghi G, Giani A. Reproducibility of Vessel Density, Fractal Dimension, and Foveal Avascular Zone Using 7 Different Optical Coherence Tomography Angiography Devices. *Am J Ophthalmol*. 2018;186:25–31. 29169882 10.1016/j.ajo.2017.11.011

[44] Dong J, Jia Y-D, Wu Q, et al Interchangeability and reliability of macular perfusion parameter measurements using optical coherence tomography angiography. *Br J Ophthalmol*. 2017;101(11):1542–1549. 28336674 10.1136/bjophthalmol-2016-309441 PMC5800775

[45] Chung CS, Nesper PL, Park JJ, Fawzi AA. Comparison of Zeiss Cirrus and Optovue RTVue OCT Angiography Systems: A Quantitative and Qualitative Approach Examining the Three Capillary Networks in Diabetic Retinopathy. *Ophthalmic Surg Lasers Imaging Retina*. 2018;49(11):e198–e205. 30457656 10.3928/23258160-20181101-18 PMC6376868

[46] Lauermann J, et al Prevalences of segmentation errors and motion artifacts in OCT-angiography differ among retinal diseases. *Graefes Arch Clin Exp Ophthalmol*. 2018;256(10):1807–1816. 29982897 10.1007/s00417-018-4053-2

[47] Wessel MM, Aaker GD, Parlitsis G, Cho M, D’Amico DJ, Kiss S. Ultra–Wide-Field Angiography improves the detection and classification of diabetic retinopathy: *Retina*. 2012;32(4):785–791. 22080911 10.1097/IAE.0b013e3182278b64

[48] Shimizu K, Kobayashi Y, Muraoka K. Midperipheral Fundus Involvement in Diabetic Retinopathy. *Ophthalmology*. 1981;88(7):601–612. 6167923 10.1016/s0161-6420(81)34983-5

[49] Oishi A, Hidaka J, Yoshimura N. Quantification of the Image Obtained With a Wide-Field Scanning OphthalmoscopeImage With Wide-Field Scanning Ophthalmoscope. *Invest Ophthalmol Vis Sci*. 2014;55(4):2424–2431. 24667862 10.1167/iovs.13-13738

[50] Silva PS, Cavallerano JD, Tolls D, et al Potential Efficiency Benefits of Nonmydriatic Ultrawide Field Retinal Imaging in an Ocular Telehealth Diabetic Retinopathy Program. *Diabetes Care*. 2014;37(1):50–55. 23939541 10.2337/dc13-1292

[51] Soliman AZ, Silva PS, Aiello LP, Sun JK. Ultra-wide Field Retinal Imaging in Detection, Classification, and Management of Diabetic Retinopathy. *Semin Ophthalmol*. 2012;27(5–6):221–227. 23163280 10.3109/08820538.2012.708812

[52] Russell JF, Shi Y, Hinkle JW, et al Longitudinal Wide-Field Swept-Source OCT Angiography of Neovascularization in Proliferative Diabetic Retinopathy after Panretinal Photocoagulation. *Ophthalmol Retina*. 2019;3(4):350–361. 31014688 10.1016/j.oret.2018.11.008 PMC6482856

[53] Musgrave I, Marley P, Majewski H. Pertussis toxin does not attenuate alpha 2-adrenoceptor mediated inhibition of noradrenaline release in mouse atria. *Naunyn Schmiedebergs Arch Pharmacol*. 1987;336(3):280–286. 2891042 10.1007/BF00172679

